# Research progress on the effects of epidural electrical stimulation on lower extremity function in patients with spinal cord injury

**DOI:** 10.3389/fmed.2026.1861870

**Published:** 2026-07-09

**Authors:** Fei Xie, Chao Bai, Xinping Luan, Jian Xu

**Affiliations:** 1 Department of Neurosurgery, The Seventh Affiliated Hospital of Xinjiang Medical University, Ürümqi, Xinjiang, China; 2 Cerebral Palsy Center in Neurosurgery, Second Affiliated Hospital of Xinjiang Medical University, Ürümqi, Xinjiang, China

**Keywords:** brain-spine interface, epidural electrical stimulation, lower extremity function, neuromodulation, rehabilitation, spinal cord injury

## Abstract

Lower extremity motor dysfunction after spinal cord injury (SCI) represents a major determinant of long-term disability and reduced quality of life. Although conventional pharmacotherapy and rehabilitation training may partially attenuate secondary injury and preserve residual function, their capacity to restore motor networks below the level of injury remains limited. In recent years, epidural electrical stimulation (EES), through targeted modulation of lumbosacral dorsal root afferents and local interneuronal circuits, has been shown to increase the excitability of spinal motor networks and augment residual descending input. Moreover, when integrated with task-specific training, EES has been associated with improvements in lower extremity motor functions, including standing, stepping, walking, and spasticity control. This study narratively reviews the pathophysiological basis of SCI, the neural mechanisms underlying EES, its clinical efficacy, combined therapeutic approaches, individualized parameter optimization, and current limitations. Available evidence, though currently limited by small sample sizes and study heterogeneity, indicates that EES may promote functional recovery in patients with chronic incomplete SCI and in a subset of those clinically classified as having “motor-complete” SCI; however, its therapeutic benefit is influenced by the complex interaction of multiple factors, including the integrity of spared pathways, the level of injury, stimulation parameters, electrode configuration, and rehabilitation intensity. Further progress is required, particularly in multicenter randomized controlled trials, standardized outcome assessments, closed-loop programming, and long-term safety evaluation, to support the translation of EES from an investigational neuromodulation strategy into a broadly applicable, precision-oriented rehabilitation modality.

## 1 Introduction

Spinal cord injury (SCI) produces persistent deficits in motor, sensory, and autonomic nervous system function; among these, lower extremity motor impairment directly limits a patient’s capacity for transfers, standing, walking, and social participation. The pathophysiological course of SCI generally involves an initial primary mechanical insult followed by a secondary injury cascade characterized by ischemia, inflammation, oxidative stress, apoptosis, and glial scar formation (1–3).

Secondary injury not only broadens the extent of tissue damage but also narrows the period during which residual conductive pathways and local spinal networks retain plastic potential; in addition, the cellular composition of the inflammatory response is closely associated with patient age, injury severity, and the time window (2, 4). Spontaneous neurological recovery occurs predominantly within the first 3–6 months after injury, and most changes in Association Impairment Scale (AIS) grade and motor function are observed within 9 months post-injury. In patients with chronic SCI, particularly those with thoracic motor-complete injury, conventional training alone yields minimal restoration of voluntary walking capacity (5, 6).

Accordingly, a key objective in SCI rehabilitation research has been to enhance the excitability of spinal networks below the level of injury during the chronic phase and to restore the contribution of residual descending input to motor output. Traditional pharmacological interventions are directed primarily toward neuroprotection in the acute and subacute stages, whereas activity-based rehabilitation seeks to induce neuroplasticity through repetitive, weight-bearing, and task-specific input; but, its efficacy is substantially limited by injury severity (7–9).

Epidural electrical stimulation was originally introduced for chronic pain management and has been extended to motor and autonomic restoration after SCI (10). Unlike pharmacological neuroprotection or rehabilitation alone, EES aims to place spinal sensorimotor networks into a permissive excitability state in which residual descending input and task-related sensory feedback can more effectively generate motor output (10–12). In the broader field of neuromodulation, several overlapping terms such as spinal cord stimulation (SCS), epidural spinal cord stimulation (eSCS), and epidural electrical stimulation (EES) are often used; for conceptual consistency, this manuscript utilizes EES throughout to specifically denote the epidural application of electrical currents to restore function.

The narrative review question is: under what neurobiological, patient-specific, and technological conditions can EES translate residual spinal circuitry into clinically meaningful lower-extremity motor recovery? Its added value is to link mechanisms, patient selection, stimulation paradigms, and rehabilitation intensity within a single lower-extremity-centered framework.

## 2 Lower extremity dysfunction after SCI and the rationale for EES therapy

With respect to lower extremity functional recovery after SCI, the key issue is not simply whether the corticospinal tract remains anatomically intact, but whether the lumbosacral spinal network preserves an adequate structural substrate composed of “afferent–interneuron–motor neuron” circuits and whether, under exogenous modulation, this circuitry can be shifted into an excitable state capable of supporting standing or walking (11, 12).

Activity-based therapy (ABT) and weight-bearing treadmill training can improve gait in patients with incomplete SCI; however, their efficacy is substantially lower in individuals with clinically motor-complete SCI than in many animal models. This disparity suggests that human walking recovery depends strongly on residual supraspinal input, preserved proprioceptive feedback, and intact segmental circuitry below the lesion (9, 11, 13). Importantly, a motor-complete clinical classification should not be equated with anatomical transection. Some patients classified as AIS A or B may retain sparse, functionally silent descending pathways that become detectable only when spinal excitability is increased by EES and training.

Patient stratification should therefore consider injury completeness, neurological level, time since injury, lower motor neuron (LMN) integrity, muscle condition, spasticity, and the rehabilitation environment. Severe conus medullaris, cauda equina, or peripheral nerve involvement may reduce responsiveness because the motor pools and peripheral effectors required for EES-mediated recruitment may no longer be sufficiently intact (12–14). From the standpoint of technological development, EES has advanced from early tonic stimulation to task-specific spatiotemporal stimulation, percutaneous or paddle-lead implantation strategies, and individualized programming approaches (15–17).

## 3 Potential mechanisms through which EES improves lower extremity function

**FIGURE 1:**
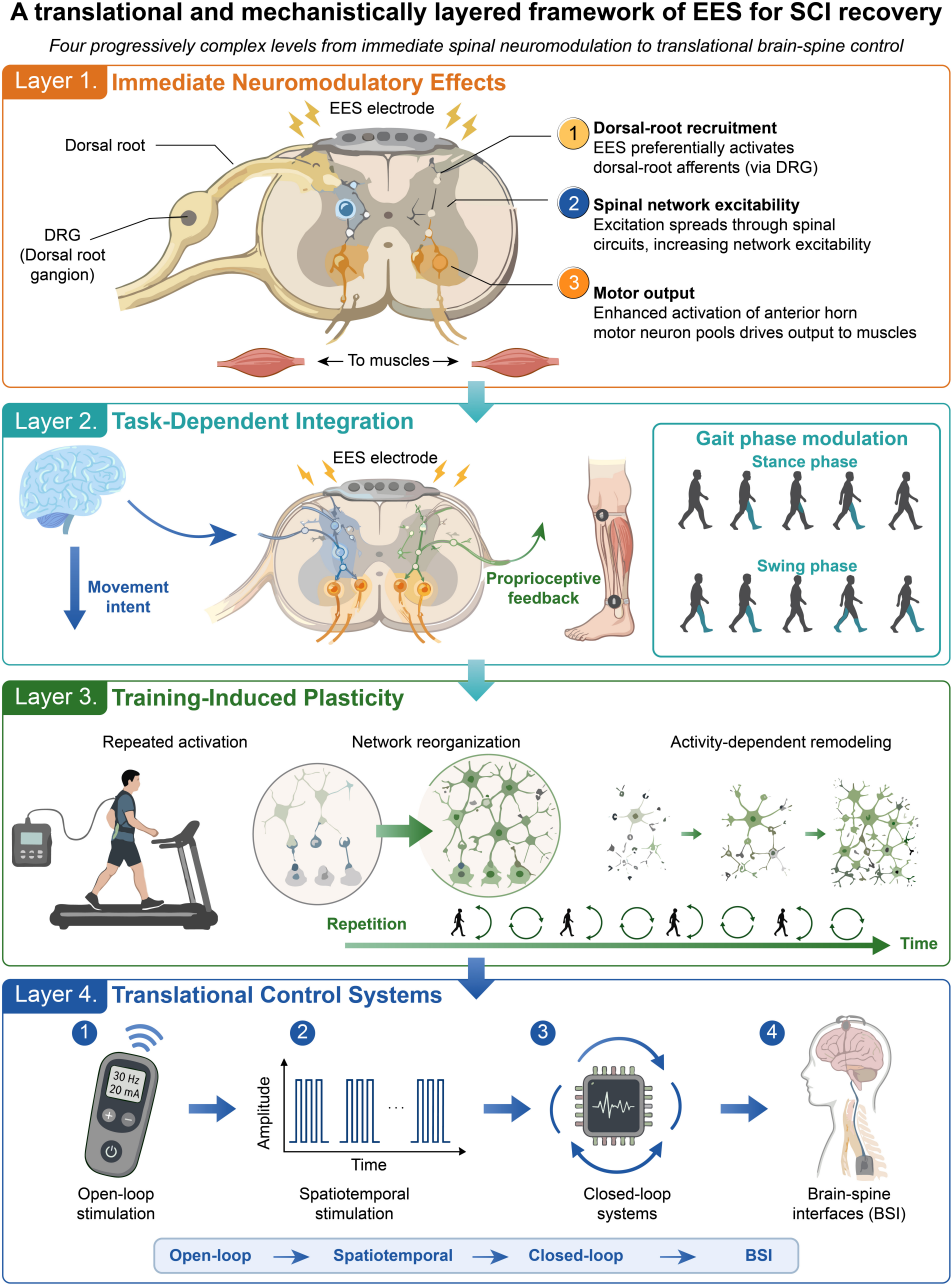
A translational and mechanistically layered framework of EES for SCI recovery. Layer 1 summarizes the immediate neuromodulatory effect of EES: epidural stimulation preferentially recruits dorsal-root afferents, increases lumbosacral network excitability, and facilitates motor output through interneuronal and anterior horn motor-neuron pools. Layer 2 illustrates task-dependent integration, in which movement intent, proprioceptive feedback, weight-bearing input, and gait-phase modulation determine whether increased spinal excitability is translated into standing or walking. Layer 3 shows training-induced plasticity: repeated pairing of stimulation with task-specific rehabilitation may promote network reorganization and activity-dependent remodeling, although direct cellular evidence in humans remains limited. Layer 4 depicts the translational evolution of EES control strategies, progressing from open-loop stimulation to spatiotemporal stimulation, closed-loop systems, and brain-spine interfaces. This Figure is intended to connect the mechanistic discussion with clinical programming, patient selection, and future neuroengineering directions.

To address the mechanistic heterogeneity of EES, its effects on lower-extremity recovery can be organized into four progressively complex layers: immediate neuromodulatory effects, task-dependent sensorimotor integration, training-induced plasticity, and translational control systems. This layered framework links the biological mechanism of dorsal-root recruitment to clinically observable functions such as standing, stepping, and walking, and also explains why EES efficacy depends on patient selection, stimulation timing, rehabilitation intensity, and programming strategy. This four-layer mechanistic and translational framework is summarized in Figure 1.

### 3.1 Layer 1: immediate neuromodulatory effects - dorsal-root recruitment, spinal network excitability, and motor output

Epidural electrical stimulation does not primarily act by directly stimulating lower-limb muscles or replacing supraspinal commands. Computational, animal, and human neurophysiological studies indicate that epidural stimulation preferentially recruits large-diameter proprioceptive afferents near the dorsal roots and dorsal columns. These afferents then activate interneuronal and motor-neuron pools through synaptic pathways, thereby shifting the lumbosacral spinal cord into a more excitable state in which otherwise weak inputs can generate measurable motor output (12, 18, 19).

This mechanism is highly segment-specific rather than a simple global activation of the spinal cord. Stimulation over roots innervating hip, knee, and ankle motor pools can produce different flexor-extensor and proximal-distal recruitment patterns, while frequency, pulse width, amplitude, polarity, and contact configuration further shape the final output. Human studies also suggest that EES-evoked recruitment may retain features of orderly motor-neuron activation under selected conditions, which may help explain why stimulation can support trainable and coordinated rather than merely synchronous movement (20, 21).

### 3.2 Layer 2: task-dependent integration - proprioceptive feedback, gait phase modulation, and movement intent

The immediate excitatory effect of EES is insufficient by itself to restore functional walking. Locomotion requires integration of stimulation with proprioceptive feedback, load-related sensory input, gait phase, posture, and voluntary or cue-driven movement intent. Continuous broad-field stimulation may increase muscle activity but can also disrupt proprioceptive signaling, including through collision of afferent signals, thereby impairing the sensory feedback required for human walking. In contrast, spatiotemporally targeted stimulation activates appropriate dorsal roots during specific gait phases and better preserves the physiological sensorimotor loop (15, 16, 22).

This task-dependent principle also clarifies why identical parameters may generate different effects during supine testing, standing, treadmill stepping, and overground walking. EES should therefore be understood as an enabling intervention: it raises the responsiveness of spinal networks so that residual descending commands, visual or auditory cues, and peripheral feedback can be converted into effective motor output. This is particularly relevant for clinically motor-complete but anatomically incomplete SCI, in which residual pathways may be functionally silent unless spinal excitability is increased (22–24).

### 3.3 Layer 3: training-induced plasticity - repeated activation, network reorganization, and activity-dependent remodeling

When EES is combined with repeated, task-specific rehabilitation, immediate stimulation-enabled movement may gradually evolve into longer-lasting functional gains. Clinical studies have reported partial recovery of voluntary movement or walking capacity after intensive EES-assisted training, while translational work suggests that recovery may involve activity-dependent selection and reorganization of spinal neuronal subpopulations involved in gait. Therefore, the main therapeutic goal is not only to produce movement during stimulation, but to repeatedly pair stimulation, intention, sensory feedback, and successful task execution in order to drive adaptive remodeling (16, 22, 25).

At the cellular and molecular level, studies of electrical stimulation and SCI models suggest possible modulation of inflammatory signaling, glial activation, neurotrophic factors, and synaptic organization. However, these mechanisms should be interpreted cautiously in human SCI because direct histological confirmation after EES is not currently feasible. Thus, the strongest human evidence supports network-level neuromodulation and training-dependent functional plasticity, whereas claims about cellular neuroregeneration remain primarily preclinical (26–28).

### 3.4 Layer 4: translational control systems - from open-loop stimulation to closed-loop and brain-spine interfaces (BSI)

The technological evolution of EES mirrors this mechanistic progression. Early open-loop stimulation increased spinal excitability in a relatively tonic manner, whereas modern paradigms use task-specific spatiotemporal patterns to coordinate stimulation with gait phases. The next translational step is closed-loop control, in which electromyography (EMG), evoked compound action potentials (ECAPs), kinematics, or other feedback signals are used to adjust stimulation in real time. BSI extend this principle further by linking cortical motor intent to spinal stimulation, allowing more natural standing, walking, stair climbing, and terrain adaptation (17, 24, 29).

## 4 Clinical evidence: restorative effects of EES on lower extremity function

Over the past decade, clinical evidence regarding EES has progressed from single-case proof-of-concept reports to multi-patient case series and, more recently, to standardized phase-based clinical investigations. Overall, the effects of EES on lower extremity function are reflected primarily in the restoration of standing, improvement in stepping and walking, enhancement of coordinated movement among the trunk, hips, knees, and ankles, and mitigation of spasticity; moreover, after prolonged training, partial neurological gains may persist even in the absence of stimulation (13, 30, 31).

Available studies further indicate that EES does not confer uniform benefit across all patients with SCI. Its therapeutic efficacy depends heavily on the presence of residual descending connections, the level and chronicity of injury, the spinal segments covered by the electrode array, the quality of stimulation programming, and the extent to which high-intensity task-specific training is incorporated (12, 17, 32). Representative clinical studies on EES and lower-extremity outcomes are summarized in Table 1.

**TABLE 1:** Representative clinical studies on epidural electrical stimulation (EES) and its effects on lower extremity function.

References	Study population (cases, Level, AIS)	Follow-up duration	Intervention	Primary outcomes	Main implications
Harkema (13)	1; C7-T1; AIS B	7 months	Continuous lumbosacral EES plus standing/stepping testing	Sustained weight-bearing standing was achieved with individualized parameters, accompanied by assisted stepping-like activity.	Established proof of concept for human EES.
Angeli (23)	4; C7:1 T2:1 T4:1 T5:1; AIS A:2 AIS B:2	3 case: 7 months; 3 cases: NR	Early postoperative testing of voluntary movement under EES	Partial voluntary lower extremity movement was restored when stimulation was activated.	Indicates that clinically “complete” injury does not necessarily signify the absence of residual pathways.
Angeli (32)	4; T4:2 C5:1 T1:1; AIS A:2 AIS B:2	>1 year	EES plus intensive gait training	Two patients Achieved overground walking, and all four achieved independent standing.	Demonstrates EES combined with training can enable functional walking.
Gill (33)	1; T8; AIS A:1	43 weeks	EES plus 43 weeks of multimodal rehabilitation	Independent stepping and overground walking were achieved with the aid of a walker and balance assistance.	Highlights the importance of prolonged task-specific training.
Wagner (16)	3; cervical region; AIS D:2 AIS C:1	5 months	Spatiotemporally targeted EES plus a pulse generator that could be triggered in real time	Overground walking control improved within 1 week, and partial recovery of movement without stimulation was observed after several months.	Advanced the paradigm of task phase-specific stimulation.
Rowald (22)	3; T4:1 T6/T7:1 T5/T6:1; AIS A:2 AIS B:1	6 years	Activity-dependent spatiotemporal neuromodulation	Rapid restoration of trunk and lower extremity motor control	Further reinforces the value of individualized sequential stimulation.
Lorach (24)	1; C5/C6; NR	>1 year	Brain-spine interface plus EES	More natural standing, walking, stair climbing, and negotiation of complex terrain were achieved.	Demonstrates the feasibility of the brain-spine interface.
Ren (34)	EES + physiotherapy (PT): 11 PT: 10;C4: 10 EES + PT 7 PT 4 T4 470; EES + PT 4 PT 6; EES + PT: AIS B: 8 AIS C: 2 AIS D: 1 PT: AIS B: 6 AIS C: 1 AIS D: 3	14 days (EES + PT); 19–25 months (all)	EES plus physical therapy versus control	Functional recovery with EES plus physical therapy was superior to that achieved with training alone.	Suggests that EES also provides additive benefit in incomplete SCI.
Chhabra (35)	5; T4:1 T5:2 T10-11:2; AIS A:3 AIS B:1 AIS C:1	35–41 months	Prospective exploratory study: sEES plus gait training	Improved weight-bearing standing control was achieved in 80% of patients, and 60% were able to walk without knee braces.	Supports larger-sample studies in Asian populations.
Romeni (36)	2; T7-T9:1 T3:1; AIS C:2	6 months	High-frequency EES plus rehabilitation	Lower extremity spasticity was reduced, and walking recovery was promoted.	Suggests dual benefits in spasticity reduction and gait recovery.
Albano (37)	1; T11)no c AIS A:1	6 months	Individualized EES treatment	Lower extremity motor control improved.	Suggests that cases involving the conus medullaris/film terminale region may also benefit.
Yao (38)	2; T9:1 T4:1; AIS B:1 AIS C:1	6 months	PET/CT evaluation after 6 months of EES	Redistribution of ^18^F-FDG was observed in the stimulated spinal cord segments and lower extremity muscles, suggesting metabolic remodeling	Provides clues for imaging biomarkers

NR, not reported or not consistently specified in the summarized information available for the present table.

### 4.1 Patient selection: incomplete SCI, clinically motor-complete SCI, injury chronicity, and LMN integrity

Patient selection should be framed as a layered biological problem rather than a binary distinction between complete and incomplete injury. In incomplete SCI, EES may amplify residual descending drive and improve the coupling between voluntary intent, proprioceptive feedback, and lumbosacral motor output. In clinically motor-complete SCI, the therapeutic window is narrower but not necessarily absent, because AIS A or B classification does not prove anatomical transection. Several EES studies have shown that some clinically motor-complete patients can generate voluntary movement or standing/walking-related output when spinal excitability is increased, supporting the concept of residual but functionally silent supraspinal-spinal connectivity (5, 23, 32).

Structural and functional biomarkers are therefore needed to refine responder selection. MRI evidence of spared tissue bridges, lesion extent, and axial preservation may help identify patients in whom descending or propriospinal pathways remain available for neuromodulatory amplification. In this context, tissue-bridge studies in cervical and thoracic SCI support the broader principle that residual spinal tissue integrity is strongly associated with neurological recovery and should be incorporated into future EES stratification models (39–41).

Lower motor neuron integrity represents another essential boundary condition. EES can modulate spinal networks only when the relevant dorsal roots, interneurons, anterior horn cells, peripheral nerves, neuromuscular junctions, and muscles remain sufficiently excitable. Severe conus medullaris or cauda equina damage, extensive root injury, profound denervation, and advanced muscle atrophy may therefore limit the conversion of stimulation into functional lower-extremity output. Conversely, recent conus medullaris experience suggests that such involvement should not be treated as an absolute exclusion criterion; rather, LMN integrity should be evaluated using clinical examination, EMG, evoked responses, imaging, and muscle status (12, 37).

Injury chronicity should also be incorporated into patient selection. Acute and subacute stages are characterized by evolving inflammation, edema, secondary injury, and spontaneous neurological recovery, whereas chronic SCI is more strongly constrained by stable tissue loss, maladaptive plasticity, spasticity, muscle atrophy, and reduced responsiveness to conventional training. For this reason, future EES trials should report injury stage, neurological level, AIS grade, residual tissue bridges, electrophysiological recruitability, LMN integrity, spasticity, muscle condition, rehabilitation dose, and stimulation maps. Such standardized reporting will make it possible to distinguish patients who are likely to benefit from neuromodulatory amplification from those in whom the segmental circuitry or peripheral effectors are no longer sufficiently recruitable (17, 42, 43).

### 4.2 Key factors influencing lower extremity recovery outcomes

From this perspective, EES outcomes should be interpreted as the product of three interacting prerequisites: preserved biological substrate, task-matched stimulation, and sufficient rehabilitation exposure. A patient with preserved descending tissue bridges but poor segmental recruitability may respond differently from a patient with robust LMN integrity but little residual supraspinal input; similarly, effective stimulation during standing may not translate directly into walking unless gait-phase feedback and training are aligned with the stimulation strategy.

First, injury severity is not the sole determinant of recovery. Some patients clinically classified as AIS A or B may exhibit trainable voluntary movement or standing capacity after EES activation, suggesting that functional quiescence within spared pathways may be more prevalent than complete structural loss in selected cases (23, 32). Nevertheless, this observation should not be generalized to all motor-complete injuries, because anatomical disruption, LMN damage, and chronic deconditioning may substantially restrict responsiveness.

Second, stimulation parameters must be matched to the functional objective. Parameters optimized for standing often differ from those required for walking, and continuous broad-field stimulation may enhance muscle activity while simultaneously disrupting proprioceptive processing or phase-dependent gait modulation (15, 17). This reinforces the need for task-specific, segmentally targeted, and outcome-guided programming.

Third, rehabilitation training is a major determinant of whether the immediate assistive effects of EES can be converted into sustained functional recovery. Prolonged, task-specific training may allow voluntary motor improvement to persist partially after stimulation is deactivated, but such carryover has been reported mainly in small cohorts under intensive protocols (16, 24).

Fourth, electrode design and implantation segment influence recruitment selectivity. Paddle electrodes, percutaneous leads, lateral versus midline contact placement, and dorsal-root entry-zone coverage may all modify recruitment of flexor/extensor and proximal/distal muscle groups (42, 44, 45). Standardized reporting of lead position and programming maps is therefore necessary for cross-study comparison.

## 5 New advances in combination therapies

### 5.1 EES combined with activity-based rehabilitation/physical therapy (PT)

At present, the combination supported by the strongest body of evidence remains EES plus ABT/PT. Standing training, weight-bearing treadmill exercise, overground walking, and gait training provide abundant sensory input to EES, allowing spinal networks in a heightened excitability state to repeatedly receive task-appropriate driving signals (9, 46, 47).

Current studies indicate that rehabilitation alone confers limited benefit in severe SCI; however, when combined with EES, substantially greater improvements may be achieved in weight-bearing standing, stepping coordination, and overground mobility (32, 33, 48). Nevertheless, training content must be aligned with the objectives of stimulation; merely increasing training volume does not represent the optimal strategy. The determining factor is whether stimulation parameters and training tasks are consistently coordinated toward the specific functional goals of standing or walking.

### 5.2 EES combined with transcutaneous spinal cord stimulation, robotics, and exoskeletons

Transcutaneous spinal cord stimulation (tSCS) offers the advantage of non-invasive screening and can be used for preoperative evaluation of neuromodulatory responsiveness, initiation of early rehabilitation, or adjunctive treatment in combination with EES (46, 47). Randomized double-blind trials suggest that the combination of tSCS with robot-assisted weight-bearing treadmill training can improve motor scores and promote walking recovery in patients with incomplete SCI (49).

Exoskeletons and robotic training systems provide highly repetitive and quantifiable gait-pattern input, thereby forming a composite intervention that integrates neuromodulation with mechanical guidance when combined with EES. Current exploratory studies and case reports indicate that this strategy may increase training intensity, improve standing and walking performance, and reduce the need for manual assistance (49–51).

### 5.3 EES combined with brain-machine (BMI)/BSI

Brain-machine interfaces and BSI are designed to translate cortical motor intent directly into control signals for spinal cord stimulation, thereby reducing dependence on residual somatic compensatory movements. Non-invasive BMI training alone has already demonstrated the capacity to promote sensorimotor recovery in some patients with chronic SCI; on this basis, its integration with EES may enable more natural and voluntary motor control (52, 53).

Lorach et al. used a fully implanted BSI to achieve more natural standing and walking in patients with chronic tetraplegia; partial functional improvements were also observed after the system had been deactivated, suggesting that this form of intent-driven EES not only enhances immediate motor performance but may also promote long-term neural plasticity (54).

### 5.4 EES combined with molecular/regenerative therapies and closed-loop intelligent systems

Regenerative medicine strategies are intended to reconstruct connectivity across the injury site through the use of scaffolds, cells, neurotrophic factors, or gene therapy, whereas EES can function as an amplifier of neural networks below the injury; in theory, these two approaches are complementary (30, 55). Animal studies have shown that, when EES is combined with regenerative therapies, synaptic reorganization below the injury is more extensive and gait recovery is greater.

A major direction for future development lies in closed-loop intelligent systems. One category consists of neuro-engineered closed-loop platforms that adjust stimulation parameters in real time on the basis of EMG, ECAP, and kinematic signals; the other comprises multimodal systems integrating EEG, EMG, robotics, and behavioral sensors to achieve gait control that more closely approximates physiological patterns (27, 56).

These combination strategies are included here only insofar as they directly modify lower-extremity recovery, programming, or translational scalability of EES. They should not be interpreted as replacing the central focus of this review, which remains EES-mediated restoration or enabling of lower-extremity motor function after SCI.

## 6 Efficacy assessment and individualized programming

Available studies have clearly shown that the AIS score alone is insufficient for accurate evaluation of EES efficacy. Although scales such as AIS, Lower Extremity Motor Score (LEMS), Spinal Cord Independence Measure (SCIM), and Walking Index for Spinal Cord Injury II (WISCI II) can reflect macroscopic functional changes, they have limited capacity to determine whether a given parameter setting has truly improved flexor-extensor coordination, standing stability, or gait phase transition (24, 33). Accordingly, current EES research has increasingly emphasized a multilevel assessment framework.

### 6.1 Integrated assessment using clinical, neuroelectrophysiological, and imaging modalities

Key tools for individualized EES programming and efficacy assessment are summarized in Table 2. At the neuroelectrophysiological level, surface or needle EMG, ECAPs, spinal evoked motor potentials, and recruitment curve analysis can be used to determine whether electrode migration has occurred, whether lateralized recruitment is adequate, whether flexor-extensor recruitment corresponds to expectations, and whether LMN pathways remain sufficiently excitable (44, 45, 61).

**TABLE 2:** Key tools for individualized epidural electrical stimulation (EES) programming and efficacy assessment.

Assessment domain	Representative findings	Clinical significance
Neural recruitment patterns	EES recruits motor output predominantly through synaptic activation mediated by dorsal root afferents rather than by direct stimulation of anterior horn cells; under certain conditions, the recruitment order may approximate physiological patterns (18, 57).	Provides a neurophysiological basis for parameter optimization.
ECAP/evoked responses	ECAPs can be used to assess stimulus propagation, lateralized recruitment, and contact efficacy, making them suitable candidate indicators for closed-loop feedback (39, 44).	Contributes to real-time programming and electrode position assessment.
Electric field orientation/spatial selectivity	Alterations in contact orientation and configuration can modify proximal/distal and ipsilateral/contralateral recruitment patterns (58, 59).	Supports individualized electrode design.
Standing parameter screening	The slope ratio of recruitment curves can assist in identifying extensor-dominant configurations suitable for standing (45).	Reduces empirical trial-and-error.
Task-specific programming	The same patient often requires different parameter combinations for standing, walking, and autonomic function tasks (17).	Emphasizes “one task, one parameter set.”
Structural/metabolic biomarkers	MRI and PET/CT can assist in predicting and tracking therapeutic efficacy from the perspectives of structural integrity and metabolic redistribution (35, 37).	Favors patient selection and longitudinal follow-up.
Animal/large-animal platforms	Multi-electrode plus EMG platforms are well-suited for long-term, fine-grained parameter studies (60).	Provides preclinical support for clinical translation.
Responder selection and LMN integrity	AIS grade should be combined with MRI evidence of spared tissue, EMG/evoked-response evidence of segmental recruitment, and clinical assessment of denervation or severe muscle atrophy (12, 37, 43).	Improves selection of patients whose lumbosacral circuits and peripheral effectors remain recruitable.

At the imaging level, MRI can assist in evaluating residual tissue bridges, the extent of axial structural disruption, and the potential for functional recovery; metabolic imaging modalities such as PET/CT may reflect stimulation-induced metabolic redistribution within the spinal cord and muscles (35, 37). In severe SCI, the integration of structural, functional, and metabolic indices may be more effective than reliance on a single scale for identifying potential responders.

### 6.2 Individualized parameter selection

Individualized programming remains a major bottleneck in the clinical translation of EES. Substantial interindividual variability exists in the optimal combination of frequency, pulse width, amplitude, polarity, and stimulation timing; moreover, even within the same patient, the parameters required often differ according to the specific task, such as standing, walking, voiding, or blood pressure regulation (17).

At present, promising strategies include closed-loop programming using ECAPs or EMG as real-time feedback, rapid parameter screening based on recruitment curves and muscle synergy patterns to identify settings appropriate for standing or walking, and imaging-based reconstruction to optimize the placement of transcutaneous leads or paddle electrodes (42, 43, 62). These approaches will ultimately determine whether EES can transition from expert-dependent manual parameter adjustment to a reproducible and scalable therapeutic modality.

## 7 Limitations and future directions

First, the current evidence base remains limited. While several studies have produced informative results, the existing literature is overwhelmingly dominated by single-case reports, small cohort case series, and non-randomized exploratory studies. The substantial heterogeneity in stimulation parameters, electrode designs, and rehabilitation intensities across these small-sample studies severely restricts the generalizability of current findings (63). Because this is a narrative rather than systematic review, the synthesis may also be influenced by publication and selection bias; therefore, the conclusions should be interpreted as a critical conceptual synthesis rather than as a formal evidence-grade recommendation. Although several studies have produced informative results, much of the available literature consists of single-case reports, case series, or single-arm prospective studies with small sample sizes, marked heterogeneity, and substantial variation in stimulation parameters and training protocols (10, 35, 48). Second, the question of which patients are most likely to benefit has not yet been fully resolved. Future investigations should incorporate more standardized structural imaging, neuroelectrophysiological metrics, and functional outcome measures to develop predictive models for responder identification, thereby distinguishing patients with complete absence of spared pathways from those who retain residual pathways that may still be enhanced (5, 17, 39). Moreover, long-term safety and accessibility remain important concerns. Implantable systems are associated with procedural risks and ongoing costs related to surgery, infection, lead migration, reprogramming, and long-term maintenance; moreover, multicenter implementation depends on a high level of coordination across equipment platforms, clinical teams, rehabilitation settings, and programming workflows (51, 60). Finally, the central focus of the field is shifting from whether movement can be restored to whether functional recovery can be achieved in a manner that is more physiological, stable, and clinically scalable. Emerging approaches, including high-frequency spasticity reduction, spatiotemporal stimulation, BSIs, transcutaneous leads, sequential tSCS-EES, and closed-loop programming, are advancing EES from proof of concept toward precision rehabilitation (24, 36, 61).

## 8 Conclusion

Epidural electrical stimulation provides a novel approach to lower extremity functional restoration after SCI that differs from conventional pharmacotherapy and rehabilitation training alone. Its central mechanism is not the replacement of neural function, but the re-establishment of coupling among residual descending commands, sensory feedback, and task-specific training through enhancement of lumbosacral spinal network excitability, thereby supporting the recovery of standing, stepping, and walking (12, 15, 22).

Overall, EES has demonstrated encouraging clinical potential, particularly in specific subsets of patients with severe chronic motor dysfunction when integrated with specialized rehabilitation programs. However, these conclusions must be tempered by the current lack of large-scale, multicenter randomized controlled trials. Its therapeutic efficacy remains dependent on careful patient selection (particularly LMN integrity), individualized parameter programming, and sustained task-specific training. Future progress in multicenter clinical trials, closed-loop neuroengineering, and regenerative therapies will likely determine whether EES can be established as a standardized strategy for restoring lower extremity function in patients with SCI (32, 35, 61). Accordingly, current evidence supports cautious, patient-specific optimism rather than broad generalization of EES efficacy across all SCI phenotypes.
